# Measurement properties of the Brazilian version of the Pediatric Quality of Life Inventory (PedsQL™) cancer module scale

**DOI:** 10.1186/1477-7525-6-7

**Published:** 2008-01-22

**Authors:** Ana C Scarpelli, Saul M Paiva, Isabela A Pordeus, Maria L Ramos-Jorge, James W Varni, Paul J Allison

**Affiliations:** 1 Department of Pediatric Dentistry and Orthodontics, Faculty of Dentistry, Federal University of Minas Gerais – Av. Antônio Carlos 6627, Belo Horizonte, MG, 31270-901, Brazil; 2 Faculty of Dentistry, McGill University, 3640 University Street, Montreal, QC, H3A 2B2, Canada; 3 Department of Pediatrics, College of Medicine, Department of Landscape Architecture and Urban Planning, College of Architecture, Texas A&M University, 3137 TAMU – College Station, TX, 77843-3137, USA

## Abstract

**Background:**

The use of health-related quality of life (HRQOL) measurements has been increased progressively in health surveys. These measurements document the functional and psychosocial outcomes of health conditions and complement clinical indicators to provide a comprehensive description of individuals and populations' health. The Pediatric Quality of Life Inventory™ (PedsQL™) is a promising instrument with age-appropriate versions. The objective of the current paper was to evaluate the psychometric properties of the PedsQL™ 3.0 Cancer Module cross-culturally adapted for use in Brazil.

**Methods:**

A cross-sectional study was developed with 190 Brazilian families of individuals from 2 to 18 years of age, of both genders, with cancer in various phases of treatment or control. Subjects were recruited by means of convenience samples from the Pediatric Hematology/Oncology Centers at two public hospitals. 'In-treatment' status was defined as individuals who were receiving medical care to induce remission. 'Off-treatment' status was defined as individuals for whom all therapy was completed for a period of at least one month. Reliability was determined through test-retest reliability and internal consistency. The validity of the Cancer Module was determined through discriminant and convergent validity. Correlations between the scores obtained by the children/adolescents with cancer and their guardians were assessed.

**Results:**

Test-retest reliability demonstrated good correlation (0.69–0.90 for children/adolescents; 0.71–0.93 for guardians) and adequate agreement of the items (0.26–0.85 for children/adolescents; 0.25–0.87 for guardians). Internal consistency demonstrated adequate indices in comparisons between groups (α = 0.78–0.80 for children and adolescents; 0.68–0.88 for guardians). The 'pain and hurt', 'nausea', 'procedural anxiety' and 'treatment anxiety' subscales proved capable of distinguishing the groups of children in treatment and off treatment (p < 0.05). Positive significant correlations were observed between the scores of the PedsQL™ 3.0 Cancer Module and the PedsQL™ 4.0 Generic Core scales. Weak correlations were found between the reports of the children and those of the guardians.

**Conclusion:**

The Brazilian version of the PedsQL™ 3.0 Cancer Module exhibited good measurement properties regarding reproducibility and construct validity.

## Background

Childhood cancer represents from 0.5 to 3.0 percent of malignant tumors in the world. In Brazil, the estimated incidence of children with tumors in 2006 was 2.5 percent of all cases of malignant neoplasms (11,800 individuals in the 0 to 18-year-old age group). The significant progress in anti-neoplasm therapy has led to a reduction in mortality rates in the last 40 years. Currently, 50 to 70 percent of pediatric cancer patients can be cured if diagnosed and treated early [1,2]. As a result of this increased survival rate, there have been a growing number of studies assessing health-related quality of life (HRQOL) in pediatric patients with cancer both during and following treatment [1,3,4].

Disease-specific HRQOL assessment instruments have been developed to determine the impact of disease and treatment on the quality of life of patients. Moreover, decisions for the implementation of improvements in public healthcare may be adopted based on the impact of interventions on quality of life [1]. However, there are a limited number of instruments designed to measure the HRQOL of pediatric patients with cancer [5-7]. Research carried out on the Medline database involving studies from 1950 to 2006 and using the descriptors 'neoplasms', 'quality of life', 'questionnaire' and 'children' revealed 193 published articles. An analysis of these publications identified three disease-specific instruments for pediatric cancer (Pediatric Quality of Life Inventory™ (PedsQL™) 3.0 Cancer Module, Quality of Life in Childhood Cancer, and the Minneapolis-Manchester Quality of Life Instrument), none of which had yet been translated and validated for Brazilian Portuguese.

The decision was made to use the PedsQL™ 3.0 Cancer Module to assess the impact of cancer on the HRQOL of children and adolescents. The PedsQL™ 3.0 Cancer Module is disease-specific HRQOL instrument developed to measure the impact of symptoms and treatment on the quality of life of pediatric patients with cancer. This decision was based on the fact that it is a multidimensional, cancer-specific instrument of easy comprehension and designed for pediatric patients between the ages of 2 and 18 years. Furthermore, it is available in a self-report version designed for children/adolescents and a proxy-report version for guardians.

The aim of the present study was to test the psychometric properties of the PedsQL™ 3.0 Cancer Module cross-culturally adapted to Brazilian Portuguese.

## Method

### Target population

The present validation study was developed in the city of Belo Horizonte, Minas Gerais, Brazil, from August through November 2006. The city is located in the central southern region of the state. It has an extension of 330.93 km^2 ^and 100% of the population resides in urban areas (2,238,526 inhabitants).

Subjects were recruited by means of convenience samples from the Pediatric Hematology/Oncology Centers at two public hospitals of the city. A total of 190 families of Brazilian children between the ages of 2 and 18 years, of both genders, with malignant neoplasm in various phases of treatment or control of the disease participated in the study. 'In-treatment' status was defined as individuals who were receiving medical care to induce remission (n = 140, 73.7%). 'Off-treatment' status was defined as individuals for whom all therapy was completed for a period of at least one month (n = 50, 26.3%) [1]. The existence of another illness or concomitant syndrome to the malignant neoplasm was established as an exclusion criterion. The choice of age group was determined by the targeted age range of the selected instrument.

The instruments were applied to pediatric patients between the ages of 5 and 18 years (n = 124). Twelve children did not answer the questionnaires. All guardians (88.4% parents, 11.6% others) answered the instruments (n = 190) reporting on the quality of life of children. Children between the ages of 2 and 4 years (n = 54) did not answer the questionnaires, as consistent with the instrument requirements. All guardians (88.4% parents, 11.6% others) answered the questionnaires (n = 190) reporting on the quality of life of children. Patients and guardians present at the hospitals on the days scheduled for the interviews were selected to participate in the study. The PedsQL™ 3.0 Cancer Module 3.0 and PedsQL™ 4.0 Generic Core Scales were administered at the hospital internment units (n = 35, 18.4%) and the outpatient treatment units (n = 155, 81.6%) while the families awaited medical care.

The questionnaires were administered by means of interviews with the children/adolescents as well as the guardians, who were interviewed separately. During the interviews, the guardians also responded to a form regarding information on age, family relation and degree of schooling. In order to characterize the families in economic terms, the Brazilian Economic Classification Criteria was used as the standard of segmentation of the population into economic classes. It is composed of five levels (A, B, C, D, E), for which A is the highest and E the lowest. The goal of this classification system is to estimate the buying power of each family, as measured by the quantity of products each family can afford [8].

Interviews were performed individually by the researcher in a room reserved specifically for this end. Prior to the interviews, authorizations were obtained from the Research Ethics Committees of the institutions involved. Terms of informed consent were also obtained from the participants.

### Instruments

The Pediatric Quality of Life Inventory™ (PedsQL™) 3.0 Cancer Module is a multidimensional instrument developed by Varni et al. [9] to assess the impact of the disease and treatment on the HRQOL of pediatric cancer patients. The instrument was developed in versions for individuals in the following age groups: 5–7, 8–12 and 13–18 years; as well as for the guardians of individuals in the following age groups: 2–4, 5–7, 8–12 and 13–18 years. There is no self-report version for children between the ages of 2 and 4 years. It is structurally composed of 27 items distributed among 8 subscales: pain and hurt (2 items), nausea (5 items), procedural anxiety (3 items), treatment anxiety (3 items), worry (3 items), cognitive problems (5 items), perceived physical appearance (3 items) and communication (3 items). The scale has five Likert response options, 'never', 'almost never', 'sometimes', 'often' and 'almost always' (corresponding to scores of 100, 75, 50, 25, 0). For the versions adapted to children between the ages of 5 and 7 years, there are only three response options: 'never', 'sometimes' and 'almost always' (100, 50, 0). For this age, a Face Scale was used, comprised of 3 pictures of facial expressions varying from a smiling face to a very sad face to indicate no problem/no difficulty/no pain to a lot of problems/difficulty/worst pain. Regarding the interpretation of the scale, higher scores indicate lower levels of difficulties related to the disease and/or treatment.

The PedsQL™ 4.0 Generic Core Scales was used to compare with the PedsQL™ 3.0 Cancer Module in order to evaluate its construct validity. The Generic Scale is made up of 23 items distributed among 4 subscales: physical functioning (8 items), emotional functioning (5 items), social functioning (5 items) and school functioning (5 items). It can be used in studies assessing the HRQOL healthy children and adolescents and pediatric patients with acute and chronic health conditions. It is available in versions for children in the age groups 5–7, 8–12 and 13–18 years; as well as the guardians of the children in the age groups: 2–4, 5–7, 8–12 and 13–18 years. As with the PedsQL™ 3.0 Cancer Module, the scale is made up of five Likert response options. Regarding interpretation, three scores can be obtained: the total score; the score referring to physical health (score of the physical functioning subscale); and the score referring to psychosocial health (combined scores of the emotional functioning, social functioning and school functioning subscales). Higher scores indicate a better quality of life.

### Statistical analysis

Test-retest reliability was determined through the calculation of the Intraclass Correlation Coefficient (ICC) regarding the scores of the 8 subscales of the PedsQL™ Cancer Module. 95% confidence intervals were estimated. The Intraclass Correlation Coefficient was measured according to the following values: ≤0.40 weak correlation; 0.41–0.60 moderate correlation; 0.61–0.80 good correlation; and 0.81–1.00 excellent correlation [10,11]. A scale with ordered categories implies that disagreement between different pairs of categories signifies different levels of seriousness depending on their position in the sequence. The Weighted Kappa Coefficient (kw) was also calculated for each question of the instrument to measure the degree of agreement of each pair of observations. The criteria described by Landis & Koch [12] were considered in the interpretation of agreement: -1.0 to 0.0 poor; 0.0 to 0.20 discrete; 0.20 to 0.40 regular; 0.40 to 0.60 moderate; 0.60 to 0.80 substantial; 0.80 to 1.00 nearly perfect. The PedsQL™ Cancer Module instrument was administered twice by the same researcher to 50 study participant families (26.3% of the overall sample), with an interval of 7 days between applications.

Values regarding the internal consistency of the PedsQL™ 3.0 Cancer Module total scale score and subscales were estimated by means of Cronbach's Alpha Coefficient. Values ≥ 0.70 were considered acceptable for comparisons between groups [13-15]. Spearman's Correlation Coefficient was calculated to assess the correlation of each item with its respective subscale. Corrected Item-Total Correlation Coefficients were obtained, considering values ≥0.20 as acceptable [16].

Discriminant validity of the PedsQL™ 3.0 Cancer Module was determined by means of a comparison between the scores determined by the known groups approach (patients in treatment and off treatment). Patients in treatment were hypothesized to demonstrate lower scores on the 8 subscales of the PedsQL™3.0 Cancer Module than patients off treatment, signifying greater difficulties and limitations due to the disease and treatment [15]. The Mann-Whitney test was utilized for the analysis of this hypothesis.

Construct validity was assessed by means of correlation analysis between the subscale scores of the PedsQL™ 3.0 Cancer Module and the scores of the PedsQL™ 4.0 Generic Core Scale Computing the inter-correlations among scales provides initial information on the construct validity of an instrument [14]. We hypothesized that greater cancer-specific symptoms or problems would be correlated with lower overall generic HRQOL based on the conceptualization of disease-specific symptoms as causal indicators of generic HRQOL [17]. Spearman's Correlation Coefficient was utilized in these analyses.

The correlation between the scores obtained on the versions applied to the children/adolescents and those applied to the guardians was determined through correlation coefficients. The Intraclass Correlation Coefficients (ICC) were computed. The SPSS for Windows (version 12.0) and Microsoft Excel software programs were used for the data analysis.

## Results

### Characterization of the sample – descriptive analysis

The study involved a sample totaling 190 individuals and their families in accordance with the inclusion criteria. Distribution per age group proved to be uniform (2–4, 28.4%; 5–7, 22.1%; 8–12, 29.0%; 13–18, 20.5%) and 65.8% of the children/adolescents were male. The average age of the guardians was 35.6 years (standard deviation = 9.6); 76.3% were mothers and 65.7% had up to 8 years of schooling. Most of the families belonged to the less privileged economic levels; 53.6% pertained to Class C and 35.7% pertained to Classes D and E (low economic level) (Table 1).

**Table 1:** Descriptive analyses: demographic characteristics of the sample

Demographic characteristics	Child/Adolescent on treatment (n = 140)		Child/Adolescent off treatment (n = 50)		Total sample (n = 190)	
***Child/Adolescent characteristics***	n	%	n	%	n	%
	
**Ages (years)**						
2–4	46	32.9	8	16.0	54	28.4
5–7	32	22.8	10	20.0	42	22.1
8–12	34	24.3	21	42.0	55	29.0
13–18	28	20.0	11	22.0	39	20.5
**Gender**						
Boys	90	64.3	35	70.0	125	65.8
Girls	50	35.7	15	30.0	65	34.2
***Guardians characteristics***						
**Ages (years)**						
18–28	37	26.4	7	14.0	44	23.2
29–34	29	20.7	22	44.0	51	26.8
35–39	38	27.2	6	12.0	44	23.2
40–79	36	25.7	15	30.0	51	26.8
**Relationship to patient**						
Mother	109	77.9	36	72.0	145	76.3
Father	17	12.1	6	12.0	23	12.1
Others (brother/sister, grandmother/grandfather, aunt/uncle)	14	10.0	8	16.0	22	11.6
**Level of schooling**						
≤ 8 years	92	65.7	32	64.0	124	65.3
> 8 years	48	34.3	18	36.0	66	34.7
**Economic level**						
high (A, B)	15	10.7	5	10.0	20	10.5
intermediate (C)	75	53.6	21	42.0	96	50.5
low (D, E)	50	35.7	24	48.0	74	39.0

All guardians (n = 190) answered the questionnaires. Regarding individuals between the ages of 5 and 18 years, 12 (6.3%) did not participate in the study; ten of these (5.3%) were in the 5–7-year-old age group and two (1.0%) were in the 8–12-year-old age group. The following were the reasons given for refusing to participate: five (3.7%) did not wish to answer the questionnaires; and seven (5.1%) did not have the physical capacity necessary to answer the questionnaires (individuals with malignant neoplasms in the Central Nervous System and individuals in the terminal phase). In such cases, only the guardians participated in the study. One female adolescent with a syndrome associated with malignant neoplasm was excluded from the study.

### Reliability

Table 2 displays the values obtained during the test-retest reliability analysis regarding the PedsQL™ 3.0 Cancer Module subscales. Considering the reports of the children/adolescents, all subscales except 'nausea' exhibited excellent correlation with the Intraclass Correlation Coefficient values (>0.80). Correlation among the guardians ranged from good to excellent, with values >0.70. Agreement of the items revealed Weighted Kappa Coefficient values of 0.26–0.85 for the children/adolescents and 0.25–0.87 for the guardians, thereby ranging from regular to nearly perfect.

**Table 2:** PedsQL™ 3.0 Cancer Module: Test-retest Reliability according to versions designed for children/adolescents and guardians

PedsQL™ Subscales	Report of child/adolescent (n = 32)	Report of guardian (n = 50)
**Pain and hurt**	0.86 (0.72–0.93)*	0.71 (0.48–0.83)*
Item 1	0.39^#^	0.87^#^
Item 2	0.77^#^	0.82^#^
**Nausea**	0.69 (0.36–0.85)*	0.86 (0.72–0.92)*
Item 1	0.43^#^	0.49^#^
Item 2	0.39^#^	0.25^#^
Item 3	0.38^#^	0.30^#^
Item 4	0.41^#^	0.61^#^
Item 5	0.26^#^	0.51^#^
**Procedural anxiety**	0.89 (0.77–0.94)*	0.81 (0.67–0.89)*
Item 1	0.46^#^	0.52^#^
Item 2	0.55^#^	0.49^#^
Item 3	0.66^#^	0.70^#^
**Treatment anxiety**	0.87 (0.73–0.94)*	0.85 (0.73–0.91)*
Item 1	0.59^#^	0.43^#^
Item 2	0.43^#^	0.49^#^
Item 3	0.60^#^	0.57^#^
Worry	0.84 (0.68–0.92)*	0.85 (0.73–0.91)*
Item 1	0.43^#^	0.63^#^
Item 2	0.51^#^	0.47^#^
Item 3	0.53^#^	0.52^#^
**Cognitive problems**	0.89 (0.78–0.95)*	0.75 (0.55–0.86)*
Item 1	0.32^#^	0.34^#^
Item 2	0.85^#^	0.34^#^
Item 3	0.54^#^	0.45^#^
Item 4	0.62^#^	0.38^#^
Item 5	0.36^#^	0.72^#^
**Perceived physical appearance**	0.90 (0.79–0.95)*	0.89 (0.80–0.94)*
Item 1	0.61^#^	0.67^#^
Item 2	0.69^#^	0.52^#^
Item 3	0.45^#^	0.63^#^
**Communication**	0.82 (0.63–0.91)*	0.93 (0.87–0.96)*
Item 1	0.53^#^	0.62^#^
Item 2	0.50^#^	0.67^#^
Item 3	0.63^#^	0.46^#^

*p ≤ 0.001(2-tailed) Intraclass Correlation Coefficient (ICC) – Confidence Interval 95%

^#^Weighted kappa Coefficient (kw) was calculated for each item

Internal consistency was assessed with Cronbach's Alpha Coefficient regarding the total scale and the different subscales according to the age group of the individuals. The analysis of the results revealed values greater than 0.70 for the total scale in all age groups and in both the version designed for children/adolescents as well as that designed for guardians. However, when assessing each subscale separately, the values proved heterogeneous (Table 3). The analysis of the Corrected Item-Total Correlation Coefficients for the 27 items of the scales revealed values greater than 0.20 (Table 4).

**Table 3:** Cronbach's Alpha Coefficient on the versions of the PedsQL™ 3.0 Cancer Module designed for children/adolescents and guardians according to subscales and age group

					Total sample	
					
PedsQL™ Subscales	2–4 (n = 0)	5–7 (n = 32)	8–12 (n = 53)	13–18 (n = 39)	n	α
**Child/Adolescent**						
Total scale	NA	0.81	0.72	0.80	92	0.76
Pain and hurt	NA	0.21	0.46	-0.09	124	0.20
Nausea	NA	0.76	0.42	0.63	124	0.62
Procedural anxiety	NA	0.73	0.65	0.79	124	0.72
Treatment anxiety	NA	0.68	0.37	0.73	124	0.62
Worry	NA	0.66	0.65	0.30	124	0.58
Cognitive problems	NA	0.36	0.46	0.54	92	0.50
Perceived physical appearance	NA	0.28	0.56	0.64	124	0.51
Communication	NA	0.68	0.58	0.64	124	0.63

					Total sample	
					
PedsQL™ Subscales	2–4 (n = 54)	5–7 (n = 42)	8–12 (n = 55)	13–18 (n = 39)	n	α

**Guardians**						
Total scale	0.75	0.75	0.80	0.88	94	0.84
Pain and hurt	0.33	0.64	0.36	0.65	190	0.50
Nausea	0.49	0.70	0.83	0.81	190	0.75
Procedural anxiety	0.80	0.69	0.75	0.69	190	0.77
Treatment anxiety	0.74	0.67	0.80	0.87	190	0.78
Worry	0.77	0.82	0.75	0.63	190	0.76
Cognitive problems	0.49	0.52	0.50	0.65	94	0.55
Perceived physical appearance	0.63	0.45	0.66	0.65	190	0.63
Communication	0.79	0.80	0.77	0.63	190	0.76

NA = not applicable

n = number of individuals

**Table 4:** PedsQL™ Cancer Module: assessment of Internal Consistency Reliability according to report of the child/adolescent (n = 124) and report of the guardian (n = 190)

PedsQL™ Subscales	Report of child/adolescent Item-Total Correlation	Report of guardian Item-Total Correlation	Report of child/adolescent Corrected Item-Total Correlation	Report of guardian Corrected Item-Total Correlation	Correlation between child/guardian scores (r) (n = 124)
**Pain and hurt**					0.227*
Item 1	0.73	0.83	0.39	0.60	
Item 2	0.75	0.79	0.42	0.56	
**Nausea**					0.470**
Item 1	0.61	0.77	0.37	0.64	
Item 2	0.62	0.64	0.46	0.49	
Item 3	0.63	0.74	0.50	0.60	
Item 4	0.59	0.75	0.40	0.61	
Item 5	0.58	0.65	0.44	0.47	
**Procedural anxiety**					0.324**
Item 1	0.78	0.74	0.63	0.57	
Item 2	0.83	0.86	0.74	0.75	
Item 3	0.67	0.87	0.56	0.74	
**Treatment anxiety**					0.234**
Item 1	0.63	0.74	0.43	0.56	
Item 2	0.74	0.88	0.58	0.79	
Item 3	0.84	0.86	0.64	0.73	
**Worry**					0.247**
Item 1	0.64	0.80	0.40	0.67	
Item 2	0.77	0.87	0.55	0.74	
Item 3	0.77	0.78	0.57	0.63	
**Cognitive problems**					0.169*
Item 1	0.57	0.53	0.28	0.24	
Item 2	0.58	0.67	0.29	0.44	
Item 3	0.43	0.49	0.28	0.28	
Item 4	0.68	0.66	0.48	0.46	
Item 5	0.63	0.66	0.39	0.47	
**Perceived physical appearance**					0.214*
Item 1	0.65	0.67	0.24	0.48	
Item 2	0.70	0.77	0.36	0.56	
Item 3	0.73	0.82	0.40	0.62	
**Communication**					0.200*
Item 1	0.71	0.83	0.52	0.68	
Item 2	0.83	0.88	0.67	0.76	
Item 3	0.73	0.77	0.47	0.59	

*p < 0.05, **p ≤ 0.01 – Spearman's Correlation Coefficient

### Validity

The discriminant validity of the PedsQL™ 3.0 Cancer Module was determined by comparing the scores for patients in treatment and those off treatment. Analysis was performed employing the Mann-Whitney test. According to the scores the children/adolescents obtained, the 'nausea', 'procedural anxiety' and 'treatment anxiety' subscales were capable of differentiating the two clinically distinct groups. Regarding the scores the guardians obtained, the two sample groups were differentiated by the 'pain and hurt', 'nausea' and 'procedural anxiety' subscales (Table 5).

**Table 5:** Discriminant validity: analysis of the average and median scores obtained on the PedsQL™ Cancer Module subscales by the child/adolescent and guardian according to the clinical condition of the child/adolescent

**PedsQL™ Subscales Child/Adolescent**	On treatment (n = 83)			Off treatment (n = 41)			Significance
			
	M	Median	SD	M	Median	SD	*P *value
Pain and hurt	86.7	100.0	18.5	86.3	100.0	18.1	0.727
Nausea	76.4	80.0	19.8	90.1	90.0	9.8	<0.001
Procedural anxiety	73.7	83.3	26.5	81.5	100.0	28.3	0.030
Treatment anxiety	83.6	100.0	21.2	95.3	100.0	11.8	0.001
Worry	54.8	50.0	31.2	63.4	66.7	27.3	0.154
Cognitive problems	77.9	80.0	21.0	82.5	85.0	16.8	0.322
Perceived physical appearance	79.7	83.3	22.6	80.5	83.3	25.3	0.600
Communication	78.5	83.3	26.3	79.3	83.3	25.5	0.892
			
**PedsQL™ Subscales Guardians**	On treatment (n = 140)			Off treatment (n = 50)			Significance
			
	M	Median	SD	M	Median	SD	*P *value

Pain and hurt	86.6	100.0	22.0	93.8	100.0	15.6	0.048
Nausea	79.9	90.0	22.2	91.7	100.0	14.3	<0.001
Procedural anxiety	46.3	50.0	34.7	58.2	66.7	39.5	0.035
Treatment anxiety	69.1	83.3	33.8	72.2	83.3	34.6	0.437
Worry	78.8	100.0	30.3	77.8	91.7	27.5	0.537
Cognitive problems	82.0	87.5	20.1	84.6	90.0	20.3	0.403
Perceived physical appearance	77.4	83.3	28.3	83.3	100.0	22.8	0.300
Communication	69.4	83.3	36.8	76.2	83.3	28.3	0.581

PedsQL™: Pediatric Quality of Life; M: mean; SD: standart deviation

Higher scores on the subscales of the PedsQL™ Cancer Module indicate less difficulties/limitations

The subdivision of the sample into three groups of patients in known distinct clinical conditions ('in treatment', 'off treatment' ≤ 12 months and 'off treatment' > 12 months) demonstrated that the 'nausea', 'procedural anxiety' and 'treatment anxiety' subscales were capable of distinguishing the groups. The Kruskal-Wallis and Mann-Whitney tests were used for the statistical analysis (Table 6).

**Table 6:** Kruskal-Wallis Test values: comparison between PedsQL™ Cancer Module scores for individuals on treatment and off treatment (≤ 12 months or > 12 months)

	Child/Adolescent report					Guardians report				
	
PedsQL Subscales	n	Mean Rank	Difference	Kruskal Wallis test	*P *value	n	Mean Rank	Difference	Kruskal Wallis test	*P *value
**Pain and hurt**										
On Tx_(a)_	83	63.22		0.128	0.938	141	91.72		3.541	0.170
Off Tx ≤ 12_(b)_	20	60.65				22	104.98			
Off Tx > 12_(c)_	21	61.43				27	107.52			
**Nausea**			a,c***; a,b**	15.331	0.000			a,c***	17.415	0.000
On Tx_(a)_	83	53.88				141	86.67			
Off Tx ≤ 12_(b)_	20	75.45				22	106.91			
Off Tx > 12_(c)_	21	84.24				27	132.31			
**Procedural anxiety**				4.994	0.082			a,b*	5.871	0.053
On Tx_(a)_	83	57.75				141	90.46			
Off Tx ≤ 12_(b)_	20	74.93				22	119.45			
Off Tx > 12_(c)_	21	69.45				27	102.30			
**Treatment anxiety**			a,c**	11.369	0.003				4.378	0.112
On Tx_(a)_	83	55.99				141	94.12			
Off Tx ≤ 12_(b)_	20	71.00				22	116.16			
Off Tx > 12_(c)_	21	80.14				27	85.85			
**Worry**				2.963	0.227				3.289	0.193
On Tx_(a)_	83	59.29				141	97.12			
Off Tx ≤ 12_(b)_	20	63.48				22	77.82			
Off Tx > 12_(c)_	21	74.26				27	101.43			
**Cognitive problems**				1.196	0.550				1.459	0.482
On Tx_(a)_	83	60.28				141	93.99			
Off Tx ≤ 12_(b)_	20	64.35				22	108.32			
Off Tx > 12_(c)_	21	69.52				27	92.94			
**Perceived physical appearance**				0.442	0.802				0.949	0.622
On Tx_(a)_	83	61.36				141	93.55			
Off Tx ≤ 12_(b)_	20	67.08				22	104.43			
Off Tx > 12_(c)_	21	62.67				27	98.43			
**Communication**				0.178	0.915				0.462	0.794
On Tx_(a)_	83	62.2				141	94.06			
Off Tx ≤ 12_(b)_	20	65.3				22	101.36			
Off Tx > 12_(c)_	21	61.0				27	98.24			

On Tx: in-treatment sample; Off Tx ≤ 12: off-treatment ≤ 12 months sample; Off Tx > 12: off-treatment > 12 months long-term survivor sample

*p < 0.05, **p ≤ 0.01, ***p ≤ 0.001 based on Mann-Whitney Test

Construct validity was measured using Spearman's Correlation Coefficient between the scores obtained on the 8 subscales of the PedsQL™ 3.0 Cancer Module and 1) the total score; 2) the score referring to physical health and 3) the score referring to psychosocial health of the PedsQL™ 4.0 Generic Core Scale. The values demonstrate that, despite being statistically significant, correlations were weak. Furthermore, a weak correlation was observed between the scores the children/adolescents obtained and those obtained by the guardians (0.17–0.47) (Table 7).

**Table 7:** Intercorrelations among PedsQL™ Scales: scores obtained by child/adolescent above the diagonal; scores obtained by guardian below the diagonal; correlation between scores of the child/adolescent and guardian on the diagonal

		Tot	Ph	Psy	P	N	PA	TA	W	CP	A	C
Total Score (Tot)	*r*	0.390**	0.826**	0.847**	0.351	0.413 **	0.294 **	0.296 **	0.303 **	0.326 **	0.370 **	0.272 **
		0.556										
Physical Health Score (Ph)	*r*	0.873**	0.377**	0.459**	0.262**	0.324 **	0.307 **	0.257 **	0.164	0.269 **	0.238 **	0.277 **
			0.557									
Psychosocial Health Summary Score (Psy)	*r*	0.822**	0.555**	0.299**	0.349**	0.425 **	0.208 *	0.302 **	0.352 **	0.321 **	0.441 **	0.230 *
				0.427								
Pain and hurt (P)	*r*	0.412**	0.361**	0.455**	0.227*	0.246 **	0.077	0.204 *	0.069	0.187 *	0.280 **	0.092
					0.610							
Nausea (N)	*r*	0.313**	0.277**	0.359**	0.308**	0.470 **	-0.02 0	0.323 **	0.258 **	0.216 *	0.212 **	0.164 *
						0.642						
Procedural anxiety (PA)	*r*	0.215**	0.230**	0.212**	0.186**	0.129	0.324 **	0.334 **	0.104	0.196 *	0.329 **	0.072
							0.504					
Treatment anxiety (TA)	*r*	0.322**	0.207**	0.403**	0.222**	0.150 *	0.342 **	0.234 **	0.211 **	0.210 *	0.285 **	0.280 **
								0.415				
Worry (W)	*r*	0.347**	0.228**	0.388**	0.153*	0.243 **	0.004	0.194 **	0.247 **	0.023	0.286 **	0.112
									0.339			
Cognitive problems (CP)	*r*	0.412**	0.342**	0.362**	0.156*	0.066	0.052	0.205 **	0.200 **	0.169*	0.196 *	0.318 **
										0.387		
Perceived physical appearance (A)	*r*	0.299**	0.217**	0.345**	0.188**	0.305 **	0.108	0.276 **	0.254 **	0.187 **	0.214 *	0.273 **
											0.470	
Communication (C)	*r*	0.192**	0.159*	0.204**	0.005	0.024	0.150 *	0.178 *	-0.060	0.231 **	0.257 **	0.200 *
												0.280

Correlation values between total score on the PedsQL™ Generic Core Module and subscales of the PedsQL™ Cancer Module are underlined. Correlation values between the scores of the child/adolescent and guardian are in bold type. Average measure intraclass correlation coefficients (ICC) are listed in italics below Spearman's Correlation Coefficient for child/adolescent and guardians correlation. ICC was derived using two-way fixed effects model. All correlations present significance levels when *p < 0.05 and **p ≤ 0.01 (2-tailed).

## Discussion

The incidence of childhood cancer is estimated at 100 to 150 cases per million inhabitants per year and has increased by about 12% in the last 15 years. In assessing all types of neoplasms in childhood and adolescence, a greater incidence is observed among boys [18]. In the present study, the majority of the sample (65.8%) was made up of males, which is consistent with the literature.

Assessment instruments should be reproducible over time, that is, they should produce similar results in two or more administrations to the same individual, provided that the general clinical state has not been altered. The analysis of test-retest reliability suggests the adequate stability of the instrument. The 7-day interval between interviews was important in diminishing the probability of systemic alterations in the clinical condition of the patient. It is recommended that the interval between measurements be long enough to reduce the effects of memory and short enough to diminish the likelihood of systemic alterations. Although the definition of this interval is arbitrary, a period of 2 to 14 days is considered adequate [16,19-21].

Internal consistency calculated by means of Cronbach's Alpha Coefficient for the overall scale demonstrated adequate homogeneity (α ≥ 0.70) for both the version designed for children/adolescents (α = 0.76) as well as that designed for guardians (α = 0.84). Procedural anxiety subscale presented values near to or above 0.70 in all age groups. Both the 'treatment anxiety' and 'communication' subscales exhibited values near to or above 0.70, except for the individuals in the 8–12-year-old age group. The same was observed for the 'worry' subscale for individuals in the 13–18-year-old age group. The 'pain and hurt', 'cognitive problems' and 'perceived physical appearance' subscales presented values below 0.70 regarding the accounts of the children/adolescents and those of the guardians.

It is interesting to note that the study carried out by Varni et al. [15] in San Diego and Los Angeles (USA) with 339 families of individuals between the ages of 2 and 18 years with cancer exhibited Alpha Coefficients of less than 0.70 in various subscales of the versions designed for children/adolescents. Thus, such subscales were only considered for descriptive analyses. The low internal consistency may be related to the small number of items that compose the subscale [22]. Furthermore, Alpha Coefficient values may be influenced by the level of schooling in the sample [23].

The analysis of the Corrected Item-Total Correlation proved the satisfactory homogeneity of the instrument. It is known that when the correlation coefficient is lower than 0.20 or 0.30, the item should either be rewritten or removed from the instrument [14,16].

A number of studies use discriminant validity analysis as a useful method in the differentiation of groups that are known to be distinct [1,15,24,25]. The results support the hypothesis that individuals in treatment would exhibit low scores on the PedsQL™ Cancer Module when compared to individuals off treatment. Therefore, the occurrence of illness implied limitations and difficulties.

It is important to note that the 'nausea' subscale was capable of discriminating individuals in treatment and individuals off treatment for a period of ≤ 12 months and individuals off treatment for >12 months in both the version designed for children/adolescents as well as that designed for guardians. Nausea and vomiting in the first 48 after initiating the chemotherapy treatment cycle are frequently reported by individuals afflicted with neoplasms [26].

The hypothesis was confirmed with regard to the construct validity of the PedsQL™ Cancer Module scale. Individuals in treatment had lower scores on the PedsQL™ Generic Core Module, as the occurrence of childhood cancer implies restrictions to daily living. It is known that there are frequent occurrences of infection, fatigue, anemia and nausea. Emotional disorders can also be secondary reactions to treatment or attributed to a lack of motivation. Psychological affects, such as a diminished scholastic performance or capacity for social interaction, can result in neuropsychological deficiencies attributed to the toxicity of chemotherapy or the isolation to which the individual is subjected [9,15].

The analysis of the correlation between the scores the children/adolescents obtained and those obtained by the guardians revealed a weak correlation in all PedsQL™ Cancer Module subscales. The same has been found in other studies [1,9,15]. Thus, the importance of developing instruments designed for children/adolescents is stressed, as the concept of quality of life is subjective [27-30]. It is known that children, even under the age of 5 years, are capable of describing their perceptions, emotions, feelings and thoughts [31]. Furthermore, the reports of children/adolescents and their guardians tend to be similar when referring to externally perceptible physical symptoms. However, opinions are quite distinct with regard to subjective issues [27,32].

This study presents limitations that should be recognized. One difficulty observed in studies on individuals afflicted with cancer regards the small size of the sample stemming from the low prevalence of the illness [23,24,33,34]. In order to broaden this convenience sample, the study encompassed the two largest childhood neoplasm treatment hospitals in the city of Belo Horizonte, MG, Brazil. The results will be applicable to a specific population. It should be pointed out that Brazil is a country of vast cultural diversity, which limits the generalization of results and implies the need to perform adjustments [35].

It should also be stressed that the scale was developed to be administered in the form of an interview with children in the 5–7-year-old age group and self-applied in the other age groups (8–12 years and 13–18 years) as well as with the guardians. However, due to the low level of schooling among the individuals who participated in the present study, the option was made to administer the questionnaire in the form of an interview in all cases. A number of studies have demonstrated that the mode of administration does not affect the performance of the instruments [21,30]. Nevertheless, a comparison between the interview mode of administration and self-administered mode of administration needs further investigation. Finally, there was no report by the patients or guardians of any lack of comprehension regarding the questions.

The lack of validation studies on assessment scales of the quality of life among children and adolescents with cancer in Brazil hinders comparisons with the results obtained here. Furthermore, the PedsQL™ 3.0 Cancer Module' is currently undergoing validation processes in a number of countries, which have only been concluded in Germany thus far [33].

## Conclusion

The Brazilian version of the PedsQL™ Cancer Module 3.0 presented adequate properties regarding the validity of the construct. The adequate reproducibility and good validity of the scale suggest its usefulness as a parameter in studies assessing the impact of neoplasms on the quality of life of children and adolescents. Further investigation of the Brazilian Portuguese language instrument should focus on testing sensitivity and responsiveness in longitudinal studies and providing a data comparison to healthy Brazilian children and adolescents.

## Abbreviations

HRQOL: Health-Related Quality of Life; PedsQL™: Pediatric Quality of Life Inventory™

## Competing interests

The author(s) declare that they have no competing interests.

## Authors' contributions

ACS, SMP, IAP, JWV and PJA conceptualized the rationale and design of the study. MLRJ contributed to the statistical analysis and interpretation of the data. ACS and SMP drafted the manuscript. All authors read and approved the final manuscript.
